# The Use of Optical Coherence Tomography for Gross Examination and Sampling of Fixed Breast Specimens: A Pilot Study

**DOI:** 10.3390/diagnostics12092191

**Published:** 2022-09-09

**Authors:** Hala Faragalla, Bahar Davoudi, Naama Nofech-Moses, Yeni Yucel, Kiran Jakate

**Affiliations:** 1 Department of Laboratory Medicine, Unity Health Toronto, Toronto, ON M5B 1W8, Canada; 2 Department of Laboratory Medicine and Pathobiology, University of Toronto, Toronto, ON M5S 1A8, Canada; 3 Department of Ophthalmology & Vision Sciences, University of Toronto, Toronto, ON M5S 1A8, Canada; 4 Faculty of Science, Toronto Metropolitan University, Toronto, ON M5B 2K3, Canada; 5 Keenan Research Centre for Biomedical Science in the Li Ka Shing Knowledge Institute, St. Michael’s Hospital, Toronto, ON M5B 1W8, Canada; 6 Department of Ophthalmology, St. Michael’s Hospital, Unity Health Toronto, Toronto, ON M5B 1W8, Canada

**Keywords:** optical coherence tomography, breast diseases, specimen handling

## Abstract

Thorough gross examination of breast cancer specimens is critical in order to sample relevant portions for subsequent microscopic examination. This task would benefit from an imaging tool which permits targeted and accurate block selection. Optical coherence tomography (OCT) is a non-destructive imaging technique that visualizes tissue architecture and has the potential to be an adjunct at the gross bench. Our objectives were: (1) to familiarize pathologists with the appearance of breast tissue entities on OCT; and (2) to evaluate the yield and quality of OCT images of unprocessed, formalin-fixed breast specimens for the purpose of learning and establishment of an OCT–histopathology library. Methods: Firstly, 175 samples from 40 formalin-fixed, unprocessed breast specimens with residual tissue after final diagnosis were imaged with OCT and then processed into histology slides. Histology findings were correlated with features on OCT. Results: Residual malignancy was seen in 30% of tissue samples. Corresponding OCT images demonstrated that tumor can be differentiated from fibrous stroma, based on features such as irregular boundary, heterogeneous texture and reduced penetration depth. Ductal carcinoma in situ can be subtle, and it is made more recognizable by the presence of comedo necrosis and calcifications. OCT features of benign and malignant breast entities were compiled in a granular but user-friendly reference tool. Conclusion: OCT images of fixed breast tissue were of sufficient quality to reproduce features of breast entities previously described in fresh tissue specimens. Our findings support the use of readily available unprocessed, fixed breast specimens for the establishment of an OCT–histopathology library.

## 1. Introduction

Breast cancer affects one in eight women in North America and is the most prevalent (non-skin) cancer among females [1,2]. Primary treatment is surgical, either with breast-conserving surgery (also known as lumpectomy) or mastectomy. Adjuvant treatments include radiation, chemotherapy, hormonal therapy and targeted antibody treatments [1]. Due to its high prevalence, breast cancer specimens are a mainstay of the pathology grossing room. The particularly challenging ones are well known to pathologists and pathology assistants alike, and generally involve some combination of a large specimen relative to a subtle target which evades inspection and palpation by the gross examiner [3]. Examples include: post-neoadjuvant specimens with near complete response [4], small tumors which are missed on gross exam [5] and premalignant lesions such as ductal carcinoma in situ (DCIS) [6].

OCT is a real-time, non-destructive optical imaging modality, analogous to ultrasound, but exploiting the scatter and absorption properties of infrared light rather than sound waves [7]. OCT can scan surface areas up to 100 cm^2^, penetrating 1–2 mm below the imaging surface, with spatial resolutions ranging from 10–20 microns [8,9,10]. Therefore, unlike standard breast imaging tools of mammography, ultrasound and magnetic resonance imaging, the resultant OCT image depicts the tissue architecture at the microscopic level familiar to pathologists. While OCT is an established modality in ophthalmology and cardiology, utilization in breast oncology is still being explored [9,10,11,12]. We believe its properties such as easy acquisition of high-resolution images across a broad surface area could make it a useful adjunct to gross examination and sampling of breast specimens for further microscopic examination.

Despite its potential, most pathologists remain unexposed to OCT. With respect to breast tissue, reference resources on how to recognize pathologic entities on OCT are underdeveloped, and not necessarily aimed at our specialty. Furthermore, previous studies described the OCT features of *fresh* breast tissue [13,14,15,16,17], whereas breast specimens are routinely fixed in formalin for a minimum of 6 h at the time of grossing [18]. To address these unmet needs, we imaged a high volume of formalin-fixed, unprocessed breast tissue specimens that are both available and abundant in a typical surgical pathology lab. Our objectives in this study were: (1) to characterize the OCT appearance of normal, benign and malignant breast tissue entities for pathology reference; and (2) to demonstrate that unprocessed breast specimens are a suitable tissue source for OCT–histology correlation studies and that quality OCT images can be obtained from formalin-fixed material.

## 2. Methods

This study was conducted at Unity Health Toronto, with institutional research ethics board approval (17-061). Unity Health is a university-affiliated tertiary care hospital network with a dedicated breast cancer center. The OCT device was provided and operated by employees of Perimeter Medical Imaging Inc. (Toronto, ON, Canada).

Tissue Accrual: Breast specimens which had residual tissue after final diagnosis reporting, and which were earmarked for disposal, were accrued for study over a 2-month period. All specimens were fixed in 10% neutral-buffered formalin and stored at room temperature.

Unprocessed specimens were grossly examined by a breast pathologist (H.F. and K.J.) for tumor, dense fibrous tissue or other palpable abnormality. Square-shaped tissue samples (TS) of these areas, measuring up to 2.0 cm lengthwise and up to 0.5 cm in thickness, were taken for OCT image acquisition.

OCT Scanning and Tissue Processing: TS were imaged using a portable, spectral-domain OCT system (TELESTO-II Thorlabs Inc., Newton, NJ, USA) with a broad bandwidth infrared light source, centered at 1325 nm. Each TS was immobilized against a glass imaging window, with ultrasound gel in between the tissue and the glass. A scans (1D axial lines) were acquired at a rate of 28,000 per second, resulting in about 15–25 B scans (2D depth profiles) per second. Several 2D B scans enabled a 3D cross-sectional rendering (or volume) of the TS. En face views of each TS were also available through rotation of the 3D cross-sectional volume (en face views are parallel to the surface of the specimen, whereas B scans are cross-sectional views into the depth of the tissue). Imaging sessions lasted less than 2 min. The OCT imaging probe was slightly tilted from its original perpendicular orientation with respect to the tissue surface, to prevent specular reflection from the glass surface of the specimen holder. The images had a lateral and axial resolution of 12 µm and 20 µm, respectively. The TS could be visualized up to a maximum depth of 2 mm.

The TS were serially sectioned perpendicular to the scanned surface and embedded in paraffin wax on edge. Three hematoxylin and eosin (H & E)-stained slides (4 µm thick and serially cut at 100 µm) were prepared from each paraffin block.

Histology and OCT Correlation: Histology slides were reviewed by a breast pathologist, and all benign and malignant findings recorded. After undergoing training in OCT interpretation, OCT volumes were reviewed by pathologists with the assistance of an OCT expert scientist (B.D.). Significant findings identified on histology slides were correlated with the corresponding OCT images to see if there were sufficient imaging features for recognition of the entity. Identification of normal tissue features (e.g., prominent ducts, vasculature, etc.) were used as landmarks where possible.

Image Processing and OCT Heat Map Generation: Select B scans with benign and malignant features were processed in MATLAB, version R2015b (MathWorks Inc., Natick, MA, USA) to generate heat map figures. The color coding was based on the intensity (back-scattered light) level of tissue components in the OCT images.

## 3. Results

Unprocessed breast specimens from 40 different patients, including one male, were retained for study over the accrual period. Specimen types and diagnoses (taken from the final surgical pathology report) are presented in Table 1. A total of 175 TS were taken for OCT scanning with anywhere from 2 to 12 TS per specimen, depending on the gross examination.

### 3.1. Histopathologic Review of TS

The number of TS containing invasive carcinoma and DCIS was 41 (23%) and 13 (7.4%), respectively. TS with malignant findings were all sourced from lumpectomy and mastectomy specimens performed for malignancy. Of 20 lumpectomies originally performed for malignancy, half (10) contained residual malignancy within the TS. The other half yielded benign findings only. Of the three mastectomies performed for malignancy, one showed residual DCIS and another lymphovascular invasion. Unprocessed tissue from malignant lumpectomy specimens had a high yield for malignant findings in TS, even if no residual gross tumor remained (as was the case in 13/20 lumpectomies performed for malignancy).

### 3.2. OCT Interpretation

OCT detects the backscattered light from components of tissue. The scatter varies depending on the degree to which that tissue component reflects, absorbs or transmits light. What follows are descriptions and nomenclature of the properties of light scatter signal that we used to recognize and interpret features on OCT.

Intensity: Tissue components which are highly scattering, and therefore absorb less light, appear relatively bright or hyper-reflective. Components which absorb more light and scatter less are hypo-reflective and appear dull or in various shades of gray. Components that transmit all or most light, with minimal backscattering, appear dark/black.

Texture: Homogenous texture has uniform intensity; conversely, heterogeneous texture has non-uniform intensity.

Penetration depth (PD): The deepest point in the tissue with discernible features, measured in millimeters. OCT can penetrate up to 2 mm from the scan surface in most biological tissues. Tumor generally has a reduced PD compared to normal fibroglandular or adipose tissue. This is attributed to greater light absorption by tumor cells, preventing it from traveling deeper into the tissue. PD is appreciated in cross-sectional B scans (2D depth profiles) but not in en face views.

Attenuation and Attenuation pattern: Attenuation refers to loss of light along the tissue depth, either through absorption or scattering. This is a function of tissue density and composition. Highly attenuating tissue translates into decreased PD. Attenuation pattern refers to the variation of the maximum PD across the tissue in a B scan. The maximum PD can be relatively constant across the depth of the tissue, as seen in normal adipose tissue; this is referred to as uniform attenuation pattern. On the other hand, attenuation can follow a more variable pattern, as seen in invasive tumors.

Shadowing: Observed in B scans, a dark “shadow” can be seen directly beneath a strongly hyper-reflective structure, such as calcification.

Boundary: This describes the interface between adjacent and different tissue components. Benign breast parenchyma typically shows sharply demarcated transitions between fibrous and fatty tissues. Meanwhile, boundaries between tumor and adjacent normal tissue are more ill-defined due to infiltrative disease. This property can be evaluated both in B scans and en face images.

**Figure 1:**
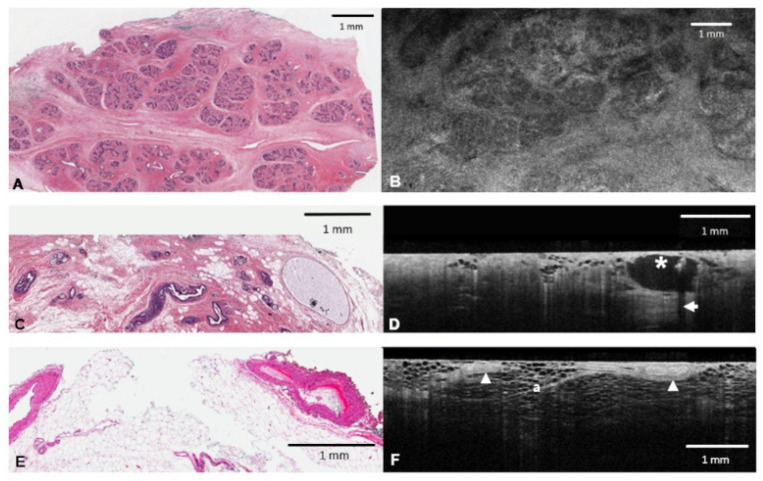
OCT appearance of benign breast tissue entities. (**A**) H & E slide of numerous duct lobules within a hamartoma. (**B**): En face OCT image of hamartoma showing hypo-reflective clusters of lobules with delicate, interdigitating stroma. (**C**): H & E slide of cystically dilated duct (on right side) containing calcification. (**D**): Corresponding, cross-sectional OCT image showing a well-demarcated cyst with hypo-reflective/black interior and hyper-reflective internal calcification (asterisk). Posterior shadow (arrow) beneath calcification. (**E**): H & E slide of arterial vessels. (**F**): Corresponding cross-sectional OCT image showing vessels (arrow heads) within adipose tissue (a). Notice bright smooth muscle layer and surrounding gray adventitia.

**Figure 2:**
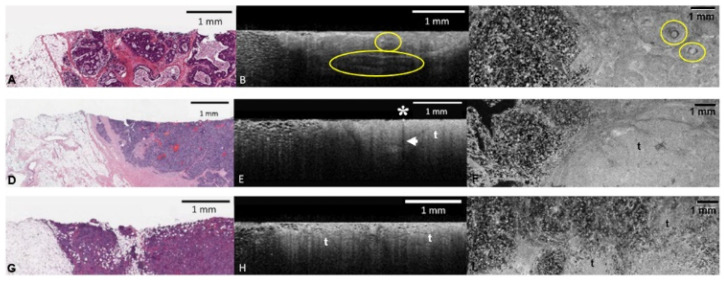
OCT appearance of malignant breast entities. (**A**): H & E slide showing conglomerate of cribriform DCIS with necrosis. (**B**,**C**): Corresponding cross-sectional (**B**) and en face (**C**) OCT images. Expanded ducts contain light gray epithelial proliferation with hyper-reflective foci of comedo necrosis (encircled). (**D**): H & E slide of invasive ductal carcinoma with pushing invasive border and clotted blood within tumor spaces. (**E**,**F**): Corresponding cross-sectional (**E**) and en face (**F**) OCT images. Tumor (t) shows dull texture and ill-defined boundary with adjacent fat. The hyper-reflective focus (asterisk) with posterior shadow (arrow) is likely clotted blood, as the tumor did not contain calcification on histology or radiology. (**G**): H & E slide of invasive ductal carcinoma infiltrating fat. (**H**,**I**): Corresponding cross-sectional (**H**) and en face (**I**) OCT images. Unlike normal fibrous tissue, the delineation between tumor (t) and fat is ill defined. Notice in 2H the irregular attenuation pattern at the lower limit of the image.

A constellation of properties was applied to recognize benign (Figure 1) and malignant (Figure 2) breast findings on OCT. OCT images demonstrated that invasive carcinoma can be differentiated from fibrous stroma based on features such as an irregular boundary, a heterogeneous texture and reduced PD. DCIS is more recognizable when comedo necrosis and/or calcification are present. Both these components are hyper-reflective and may have shadowing beneath (Figure 2). DCIS was not associated with an apparent loss of PD on B scans to the degree observed in invasive carcinoma. OCT features of benign and malignant breast entities were compiled as a reference for pathologists (Table 2). Lymphovascular invasion, seen in two TS, could not be discerned as a specific feature on OCT images. Small foci of atypical ductal hyperplasia, as well as adenosis (sclerosing or as a component of fibrocystic disease), were also not distinctive enough on OCT for characterization.

### 3.3. Heatmap Light Scatter Analysis

**Figure 3:**
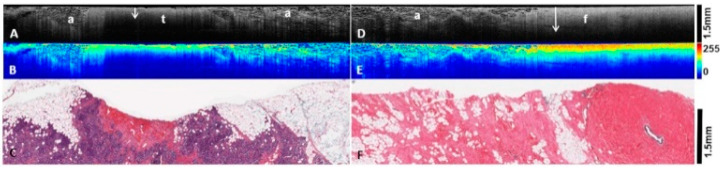
OCT image and heatmap analysis of invasive tumor (left column, **A**–**C**) and normal breast tissue (right column, **D**–**F**) from two different patients. (**A**): OCT B scans of invasive tumor (t) and surrounding adipose tissue (a). The tumors are characterized by reduced light PD (arrow) and dull texture. (**B**): Heatmap representation of corresponding OCT image above, showing lower levels of scatter by the tumor, manifested in mostly yellow hues. (**C**): Corresponding histology of invasive ductal carcinomas, high grade in 3C. (**D**): OCT B scans of normal breast tissue demonstrating fibrous stroma (f) and adipose tissue (a). The arrow shows increased light PD compared with tumors. (**E**): Heatmap representation of corresponding OCT image above. Note greater presence of warm colors and more homogenous light scattering pattern within benign fibrous tissue compared with tumors. (**F**): Corresponding histology of benign breast tissue.

**Figure 4:**
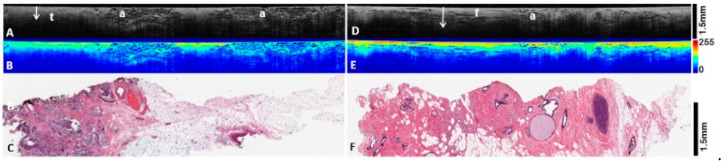
OCT image and heatmap analysis of invasive tumor (left column, **A**–**C**) and normal breast tissue (right column, **D**–**F**) from the same patient. (**A**): OCT B scans of invasive tumor (t) and surrounding adipose tissue (a). The tumors are characterized by reduced light PD (arrow) and dull texture. (**B**): Heatmap representation of corresponding OCT image above, showing lower levels of scatter by the tumor, manifested in mostly yellow hues. (**C**): Corresponding histology of invasive ductal carcinomas, intermediate grade. (**D**): OCT B scans of normal breast tissue demonstrating fibrous stroma (f) and adipose tissue (a). The arrow shows increased light PD compared with tumors. (**E**): Heatmap representation of corresponding OCT image above. Note greater presence of warm colors and more homogenous light scattering pattern within benign fibrous tissue compared with tumors. (**F**): Corresponding histology of benign breast tissue. Figure 4F also shows dilated ducts and usual ductal hyperplasia.

Heatmaps were created for select OCT images to compare the light scatter patterns of invasive carcinoma and benign breast parenchyma (Figure 3 and Figure 4). Higher tissue scattering was represented by warmer colors (red and orange) and higher intensities in the colormap (i.e., closer to 255), while lower scattering appeared in blue color and with intensities closer to zero. In Figure 3, the comparison is made using specimens from another patient in which the normal breast tissue was sourced from a prophylactic mastectomy. In Figure 4, we repeated the analysis on tumor and non-tumor tissue from the same patient.

Both heatmaps of invasive carcinoma (Figure 3B and Figure 4B) show reduced and heterogeneous light scatter when compared with their benign counterparts (Figure 3E and Figure 4E, respectively).

## 4. Discussion

Our study demonstrates the value of fixed, unprocessed breast tissue as a repository of residual pathologies, both benign and malignant. The yield of residual malignant lesions was 30% of all TS and up to 50% in TS from lumpectomies performed for malignancy. OCT images of fixed breast tissue were of sufficient quality to reproduce features of breast entities previously described only in fresh tissue specimens. Invasive carcinoma on OCT principally needs to be distinguished from normal, fibrous breast tissue. A heterogenous intensity, irregular boundary and reduced PD compared to surrounding normal tissue are the most helpful features in identifying a tumor. DCIS is most readily identified by distended ducts with comedo necrosis and calcification. Similar to traditional histopathologic criteria, the OCT features of an entity should be considered together, as a single characteristic may be insufficient for diagnosis.

The features of this technology could be useful in breast specimen sampling. Medical advances in breast cancer diagnosis and treatment have resulted in greater complexity of surgical pathology specimens [19], wherein the biopsy target can be difficult to locate on gross examination alone. Post-neoadjuvant specimens, increasing in number, may have minimal residual tumor and, in its place, a subtler tumor bed [3,4]. The combination of improved mammographic screening and greater biopsy gauge can result in sub-centimeter masses mostly removed at the time of biopsy and, as follows, imperceptible on excision [20]. Premalignant lesions, such as atypical ductal hyperplasia and DCIS, are almost always non-mass forming and, as such, grossly occult [21]. Nonetheless, the extent of DCIS requires thorough documentation by the pathologist, which is why current grossing guidelines recommend lumpectomies for DCIS and atypia be completely submitted for microscopic examination [6,22]. In fact, we find the complexity of breast specimens is often addressed by submitting greater amounts of tissue for slide review, whether this is codified in a guideline or as a mode of practice. Therefore, it is no surprise that, in a study comparing slide volume of breast and non-breast specimens, the former had almost twice the number of total slides [23].

The imaging tool most commonly used to aid in grossing breast specimens is specimen X-ray, facilitated by the arrival of portable, enclosed devices, such as the Faxitron. Specimen X-ray may be utilized by the gross examiner to identify calcification or a localization marker in order to guide tissue sampling [24]. However, these applications have their limits: mammography has been shown to underestimate the size of high-volume DCIS when compared to pathologic size estimates by a mean of 1.31 cm [25]. Thus, the extent of calcifications on specimen X-ray is not an absolute indicator of disease extent. Localization markers, such as biopsy clips and seeds, can migrate from their original target, and so their presence does not obviate the need for thorough gross examination [3]. Compared to X-ray, OCT has the benefit of providing real-time information on *microscopic* entities, rather than radiologic targets. In this way, OCT images could be used to direct tissue sampling for greater accuracy and efficiency by selecting tissue with relevant pathology. As OCT systems are both portable, non-radioactive and safe for users [9], they could be deployed directly in the grossing room by pathologists and/or pathology assistants [12]. The scanning time for large specimens is also reasonable: Schmidt et al. reported scanning complete lumpectomies in an average of 10 min with wide field OCT [10].

Before its potential in preanalytic breast pathology can be realized, OCT images need to be validated on formalin-fixed tissue, and criteria for recognizing breast entities on OCT need formulation. While fresh tissue imaged with OCT generally shows slightly better contrast than that which is formalin fixed, this does not necessarily impact interpretability [17]. In our study, we were able to reproduce OCT features of breast entities previously described in fresh tissue [13,14,15] from fixed unprocessed breast specimens. We achieved comparable PD between 1–2 mm with this tissue source [9]. The resultant images and their correlation with histology informed our base descriptions of benign and malignant breast entities (Table 2). We were also able to amplify the differences in the scatter pattern of tumor versus normal stroma by employing heat maps (Figure 3 and Figure 4), an example of how we expect OCT interpretation to be supplemented by image analysis. The reduced light scatter of tumor we observed is consistent with findings that tumors absorb light to a greater degree than normal breast tissue. One reason for this is the enhanced blood supply of breast tumors, as hemoglobin is a potent absorber of light [26].

Although not mentioned in Table 2, we sought out traditional mimickers of malignancy in our source material, such as adenosis and usual ductal hyperplasia (UDH), to learn about their OCT appearance. To this end, we examined over 30 TS with benign breast disease. Unlike a tumor mass, adenosis on OCT was indistinct, and not associated with the dramatic drop in PD or irregular attenuation pattern. Furthermore, the boundary between parenchymal stroma and fat was smooth, even in a case of florid tubular adenosis. Ducts with mild to moderate UDH were also subtle, and they resembled normal TDLUs. On the other hand, cases of florid UDH could suggest DCIS, in that they appeared as enlarged ducts expanded by light gray (hypo-reflective) epithelial content. An unlikely mimic of DCIS was dilated ducts filled with thick cellular debris, with or without calcification. In some cases, the debris, which included sloughed epithelial cells and macrophages, was hyper-reflective and had associated shadowing. The appearance approximated DCIS with comedo necrosis, and it could pose a pitfall. Further study and characterization of benign mimickers of malignancy on OCT is thus required, along with a better understanding of the false positive and false negative rates. When the latter do occur (histopathologically malignant lesions that tested negative on OCT and vice versa), mitigation strategies must be put in place. Discordant findings on histology and OCT should at least trigger a review of the OCT image by the primary pathologist, with involvement of a second reviewer and increased sampling of the specimen as further potential actions. The discordant rate between breast histopathology and OCT would need to be relatively low for this technology to improve workflow and not increase the time and complexity of processing these specimens, because the OCT findings are misleading.

## 5. Conclusions

OCT is a safe and non-destructive imaging modality with the ability to image a wide surface area, up to 2 mm in depth and with spatial resolutions high enough to depict tissue architecture and some microscopic structures. Breast pathologists should consider how this technology could be employed in their practice; we feel it shows potential as an adjunct for breast specimen grossing examination and sampling. A major limitation is that validated diagnostic criteria for breast entities on OCT are lacking, as is proficiency amongst pathologists in image interpretation. As such, we are among the first to develop an OCT reference with descriptions of breast tissue entities, to serve as a basis for further study and establishment of formal criteria.

Efforts to classify optical breast tissue images using automated algorithms are continually being developed [9]. Certainly, automated software that can quickly identify areas of interest would facilitate integration of OCT for use in the pathology lab. With such anticipated advancements, is it necessary we learn to interpret OCT? Our position is that interpretations derived from machine learning should be used as an adjunct to assist the pathologist in arriving at the final diagnosis; they should not supplant our expertise in morphology, whether the medium is histology slides or OCT.

We also recommend OCT imaging of unprocessed, fixed tissue for non-diagnostic purposes, such as OCT-histopathological correlation studies, research and validation of OCT devices and algorithms. The advantages of unprocessed tissue include its greater accessibility from a research ethics perspective, and its utilization does not interfere with specimen handling, flow of patient care or primary diagnosis.

## Figures and Tables

**Table 1:** Surgical Case Source Material for TS.

	Lumpectomy	Mastectomy	Prophylactic Mastectomy	Re-Excision	Reduction Mammoplasty
Number of specimens	26	3 ^a^	4	2 ^b^	5
Average patient age	56	51	42	69	43
Fixation time (d)	149	84	136	172	172
Invasive + DCIS ^c^	15	3	-	1	-
Invasive only	5	-	-	-	-
Atypical	2	-	1	-	1
Benign	4	-	3	1	4

D = days; ^a^ One mastectomy was post-neoadjuvant chemotherapy. ^b^ One re-excision was performed for positive margins with DCIS and the other for a new mass at the site of previously excised cancer. ^c^ Lumpectomies for DCIS alone are submitted in toto as per departmental policy; thus, this type of specimen was not available.

**Table 2:** OCT Features of Breast Histology.

Histopathologic Finding	OCT Appearance
**Benign:**	
Fibroglandular tissue	Terminal duct lobular units (TDLUs) are subtle, delicate clusters of rounded ducts, interdigitated by gray fibrous tissue (Figure 2B). The boundary with fat is sharp and well delineated.
Adipose tissue (fat)	Honeycomb pattern of dark adipocytes and bright supporting stroma (Figure 1F).
Cyst	Spherical shape with a dark lumen. Subtle gray epithelial lining may be appreciated (Figure 1D).
Vessel	Elongated tubal structures with a bright smooth muscle and slightly darker adventitia. Where present, clotted blood is strongly hyper-reflective (Figure 1F).
Calcification	Strongly hyper-reflective with posterior shadowing seen on 2D depth profiles (B scans) (Figure 1D).
**Malignant:**	
Invasive tumor	Invasive carcinoma has varied histomorphology, and this is reflected in the OCT findings. Generally, tumor intensity is duller and more heterogenous than uninvolved fibrous tissue. The boundary with adjacent fat or fibroglandular tissue is ill defined. It also exhibits reduced PD on 2D depth profiles (B scans) (Figure 2E,F).
DCIS	DCIS without comedo necrosis or calcification can be difficult to recognize on OCT. Look for groups of distending ducts which do not represent a cyst (see above). When present, comedo necrosis and calcification are both hyper-reflective, and both exhibit posterior shadowing. Cribriform architecture may be appreciated within the epithelial proliferation. The immediate stroma surrounding DCIS will appear brighter (Figure 2B,C).

## Data Availability

The datasets generated and analyzed from this study are available from the corresponding author upon reasonable request.
